# Radiological findings of breast involvement in benign and malignant systemic diseases

**DOI:** 10.1590/0100-3984.2016.0125

**Published:** 2018

**Authors:** Renato Augusto Eidy Kiota Matsumoto, Juliana Hiraoka Catani, Mirela Liberato Campoy, Arthur Magalhães Oliveira, Nestor de Barros

**Affiliations:** 1 Department of Radiology, Hospital das Clínicas da Faculdade de Medicina da Universidade de São Paulo (HC-FMUSP), São Paulo, SP, Brazil.; 2 Faculdade de Medicina da Universidade de São Paulo (FMUSP), São Paulo, SP, Brazil.

**Keywords:** Breast, Systemic diseases, Collagen disease, Lymphoma, Metastases, Mama, Doenças sistêmicas, Colagenoses, Linfoma, Metástases

## Abstract

Although the primary purpose of periodic mammograms in screening programs is to identify lesions suspected of being carcinomas, the findings are often related to systemic (benign or malignant) diseases, rather than breast cancer. Although the involvement of breast structures in systemic diseases is unusual, it can be included in the differential diagnosis of masses, skin changes, calcifications, asymmetry, and axillary lymphadenopathy. The main diagnostic entities that can be associated with such involvement are diabetes, chronic kidney disease, heart diseases, connective tissue diseases, HIV infection, lymphoma, leukemia, and metastases from primary tumors at other sites. In many cases, information related to knowledge and treatment of chronic diseases is not available to the radiologist at the time of evaluation of the mammography findings. The purpose of this essay is to offer relevant pictorial information to the general radiologist about systemic diseases involving the breast, expanding the range of differential diagnoses in order to avoid unnecessary invasive procedures.

## Abstract (pt)

Embora o objetivo primário da realização periódica da mamografia nos programas de rastreamento seja a identificação de lesões suspeitas para carcinoma mamário, muitas vezes as alterações encontradas não estão relacionadas ao câncer de mama, e sim, a doenças sistêmicas benignas e malignas secundárias de outros sítios. O envolvimento das estruturas mamárias nas doenças sistêmicas é incomum, mas pode ser incluído no diagnóstico diferencial de nódulos, alterações cutâneas, calcificações, assimetrias e linfonodomegalias axilares. As principais entidades diagnósticas que podem estar associadas ao acometimento mamário são o diabetes, a nefropatia crônica, as cardiopatias, as colagenoses, as infecções pelo vírus HIV ou parasitas, o linfoma, a leucemia e as metástases de tumores primários de outros órgãos. Muitas vezes as informações relacionadas ao conhecimento e/ou tratamento de doenças crônicas não estão disponíveis para o radiologista no momento da avaliação da mamografia. O objetivo deste ensaio é oferecer informações iconográficas relevantes a respeito de doenças sistêmicas com envolvimento mamário, permitindo ampliar o leque de diagnósticos diferenciais e evitar eventuais procedimentos invasivos desnecessários.

## INTRODUCTION

With the expansion of breast cancer screening programs, more mammographic examinations are being performed, and, as a consequence, the detection of breast findings not related to epithelial carcinomas is also more frequent. The major benign systemic diseases with radiological manifestations on mammography and breast ultrasound are diabetes, heart diseases, chronic kidney disease, HIV infection, granulomatous diseases (e.g., tuberculosis), parasitic diseases, and connective tissue diseases (e.g., dermatomyositis, scleroderma, and systemic lupus erythematosus). Within that context, patients may present, clinically, with skin changes, palpable masses and skin thickening. Malignant systemic diseases with secondary manifestations in the breasts can include lymphoma, leukemia, and metastases from primary cancer at other sites.

The initial diagnostic flow chart involves the analysis of the clinical history and previous treatments. When these tools are used in conjunction with the mammography and ultrasound findings and yet do not result in a definitive diagnosis, percutaneous biopsy can be performed. The objective of this article is to present the most common systemic diseases affecting the breasts, as well as their radiological manifestations.

## DIABETES

**Figure 1:**
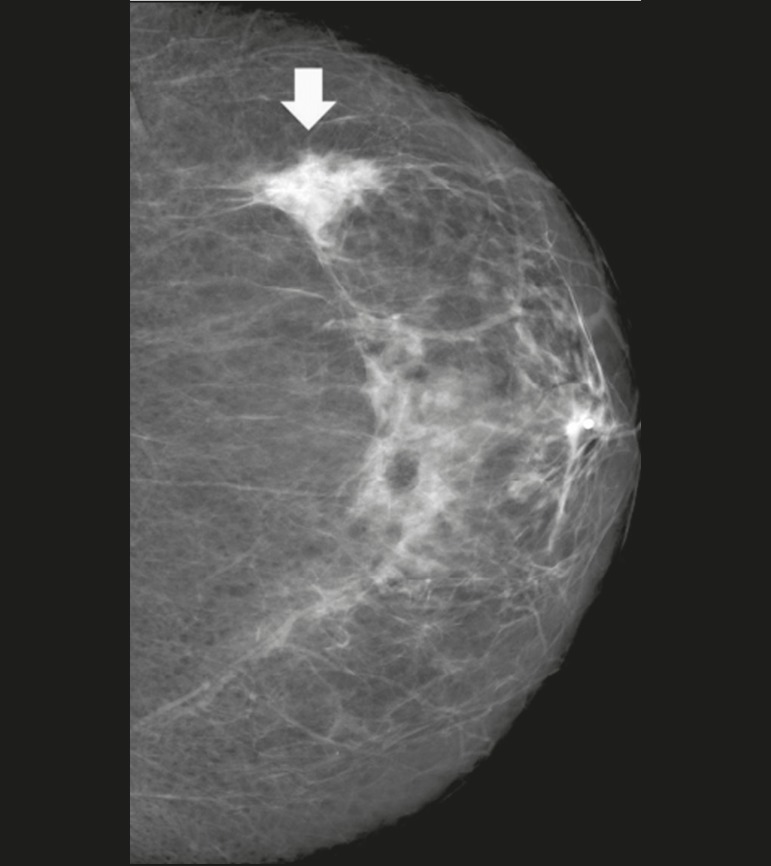
Mammogram, in a craniocaudal view, showing focal asymmetry in the upper outer quadrant of the left breast (arrow) measuring 3.0 cm, in a 46-year-old patient under insulin therapy.

**Figure 2:**
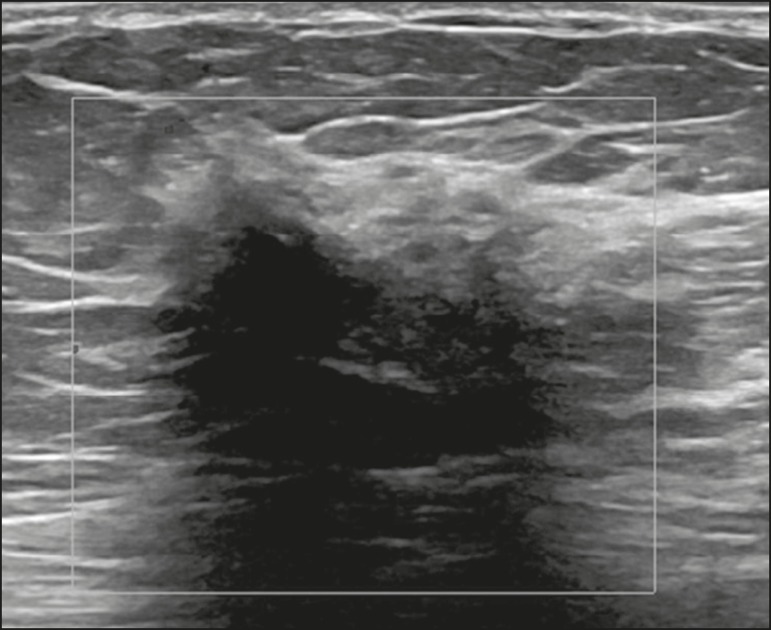
Ultrasound showing an irregular, spiculated, hypoechoic mass, with posterior acoustic shadowing, with no flow on Doppler evaluation. Percutaneous biopsy of the mass resulted in a diagnosis of perilobular lymphocytic infiltrate, consistent with diabetic mastopathy.

Diabetic mastopathy is an uncommon entity, occurring mainly in young women with a long history of type I diabetes, and affects less than 15% of insulin-dependent patients^(1)^. Although the cause is not well known, it is related to an increase in the amount of collagen, increasing the extracellular matrix in the setting of hyperglycemia^(2)^. On mammography, it manifests as focal asymmetry or a solid mass, usually in the retroareolar region, without accompanying calcifications (Figure 1). The sonographic appearance is a hypoechoic mass with indistinct or spiculated margins, with pronounced posterior acoustic shadow, and no vascularity on the Doppler evaluation^(3)^, as illustrated in Figure 2. Those presentations raise the possibility of malignancy, and, consequently, percutaneous biopsy is recommended. During the biopsy procedure, the lesion is often hard, which hampers its sampling.

## HEART DISEASES

**Figure 3:**
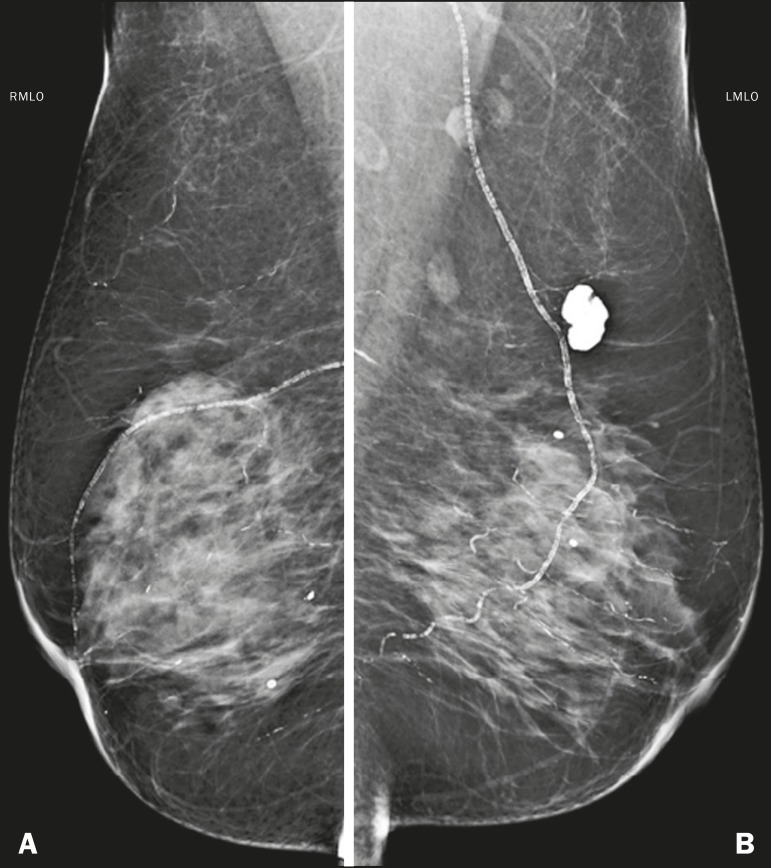
A 58-year-old female patient presenting with multiple vascular calcifications on mammography.

**Figure 4:**
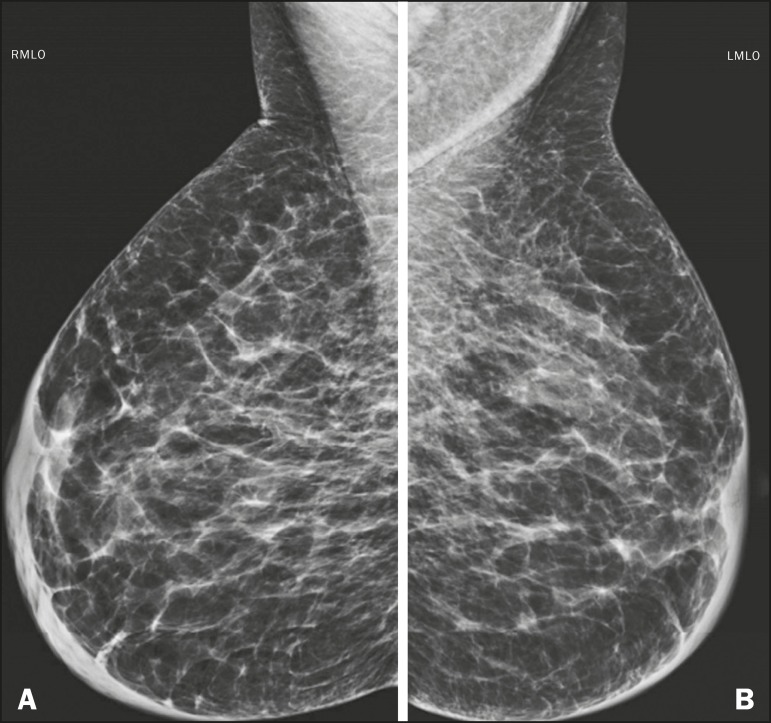
M ammogram, i n m ediolateral o blique v iews, o f a 5 7-year-old f emale patient presenting with an increase and accentuation of the trabecular breast tissue, accompanied by diffuse bilateral thickening of the skin. These findings are associated with decompensation of congestive heart failure.

There are two main aspects of heart diseases with manifestation in the breasts^(3)^: arteriopathy and edema. Arterial calcifications are common and do not cause diagnostic difficulties in mammography (Figure 3), unless they are incipient, in which case they can mimic linear suspicious calcifications. It is not well established in the literature whether the detection of arterial calcifications is related to increased cardiovascular risk. It is intuitively assumed that calcifications and peripheral arteries are a consequence of ongoing cardiovascular disease and are associated with risk factors for coronary artery disease, and this assumption is supported by some studies showing a positive association between the presence of vascular calcifications and cardiovascular disease^(4)^. As can be seen in Figure 4, the edema manifests as skin thickening, vein engorgement, and increased fibroglandular tissue density on mammography, whereas it manifests as increased echogenicity of superficial fatty planes and hypoechoic fluid collections on ultrasound^(3)^.

## CHRONIC KIDNEY DISEASE

**Figure 5:**
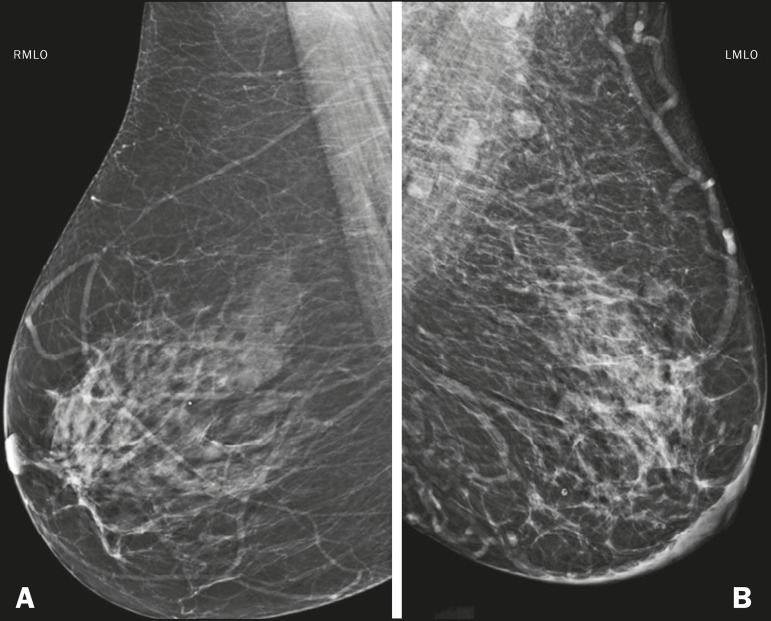
History of chronic kidney disease in the creation of a left arteriovenous fistula. Mammogram showing a vascular prominence in the left breast.

The imaging findings most commonly seen in chronic kidney diseas are related to its pathophysiology. Due to fluid retention, there are radiographic findings similar to those of congestive heart failure, with increased fibroglandular density, thickening of trabeculae, and skin thickening^(3)^. Calcifications in the medial layer of the arteries can result in prominent vascular calcifications. Secondary hyperparathyroidism can give rise to coarse, mainly cutaneous, calcifications. An arteriovenous fistula for dialysis results in prominent venous collaterals in the ipsilateral breast (Figure 5). As a consequence of the medications used in patients undergoing renal transplantation, fibroadenomas can be commonly seen in women taking cyclosporine^(5)^ and infectious processes can result from the immunosuppressive state. In men with chronic kidney disease, the drop in serum testosterone levels may cause gynecomastia.

## HIV

**Figure 6:**
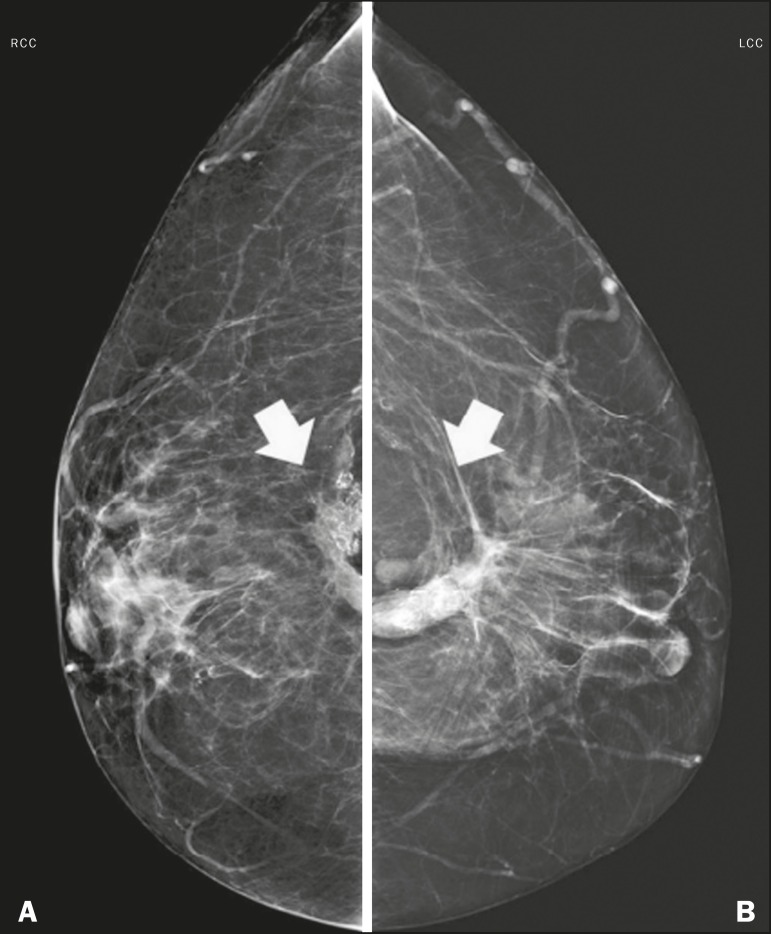
Mammogram of a 42-year-old female patient with a history of HIV infection, currently receiving antiretroviral therapy, who presented with bilateral areas of fat necrosis. The patient had a history of adipose tissue graft in the breasts due to lipodystrophy caused by HIV infection.

Axillary lymph node enlargement and infectious processes can be seen in HIV-infected individuals. The lymph nodes tend to present hyperdense and with larger dimensions, although nonspecific. On ultrasound, the lymph nodes show diffuse, symmetrical cortical thickening. Breast composition is also affected by HIV-associated lipodystrophy, because there is a lower proportion of adipose tissue, resulting in a breast with a greater density on mammography. In HIV-infected patients, there may be filling of the breast with autologous adipose material, promoting areas of fat necrosis (Figure 6).

## GRANULOMATOUS DISEASES

**Figure 7:**
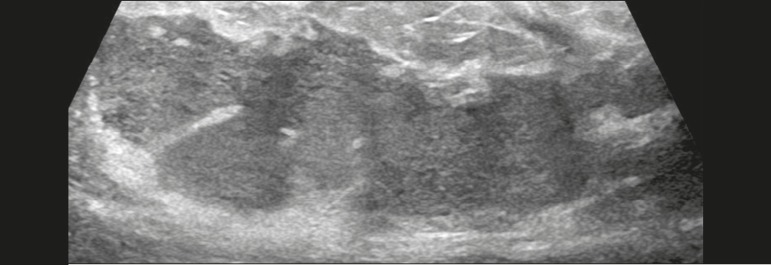
Ultrasound of a 35-year-old female patient who presented with an irregular hypoechoic collection containing some hyperechoic streaks in the central region of the left breast, which did not improve after antibiotic therapy. Sputum smear microscopy was positive for acid-fast bacilli.

Granulomatous diseases include tuberculosis and mastitis. In systemic tuberculosis, breast or axillary involvement is rare and manifests in two main forms: axillary lymphadenopathy and tuberculous mastitis. When there is lymph node involvement, the lymph nodes are enlarged, the cortex is hypoechoic, and there can be calcifications. In mastitis, ultrasound shows abscess formation represented by complex (solid-cystic) masses or fluid collections (Figure 7). Granulomas may also appear as irregular masses accompanied by edema of the adjacent fat tissue^(3,6)^. In these situations, it is difficult to make an accurate diagnosis, given that it is often impossible to exclude a malignant lesion on the basis of imaging findings alone and a biopsy is therefore necessary.

## PARASITIC INFECTIONS

**Figure 8:**
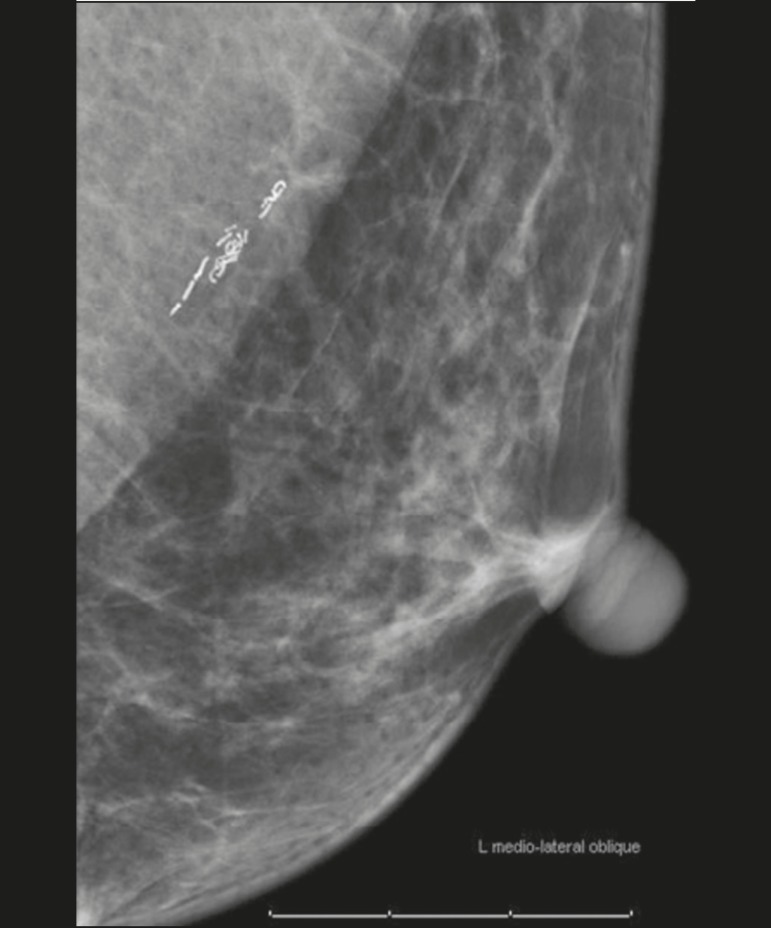
Mammogram, in a left mediolateral oblique view, of a 53-year-old female patient, under treatment for filariasis, who presenting with serpentine calcifications in the axillary tail of the breast.

Filariasis is a parasitic infection that can involve the breasts, caused by the helminth *Wuchereria bancrofti*. The main clinical manifestations occur as a consequence of obstruction of the lymphatic vessels by the presence of active or calcified worms. In the breast, the larva penetrates the lymphatic vessels and causes lymphangitis, fibrosis, and changes in the lymphatic drainage, resulting in global or focal asymmetry accompanied by trabecular and skin thickening. The larvae can later present as linear or serpentine calcifications^(7)^, as depicted in Figure 8.

## CONNECTIVE TISSUE DISEASES

**Figure 9:**
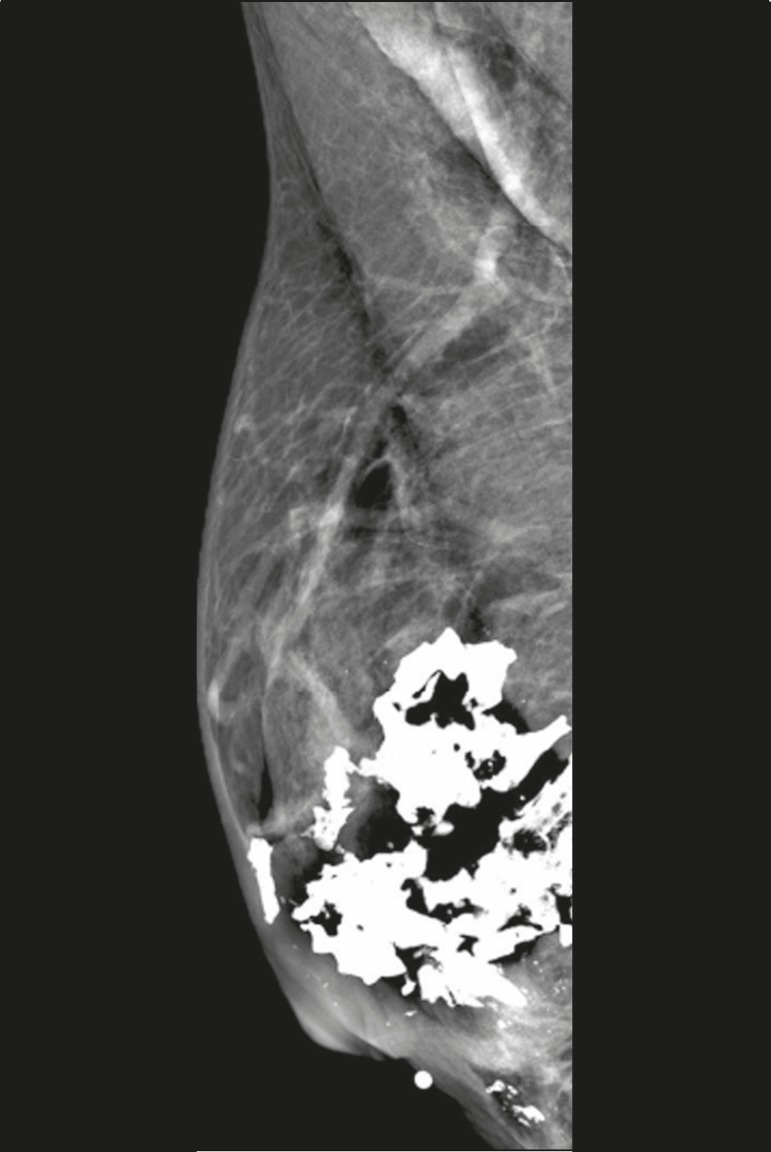
A 41-year-old female p atient, diagnosed w ith s ystemic lupus e rythematosus and under rheumatology follow-up, who presented with coarse, dystrophic calcifications in the retroareolar region of the right breast.

**Figure 10:**
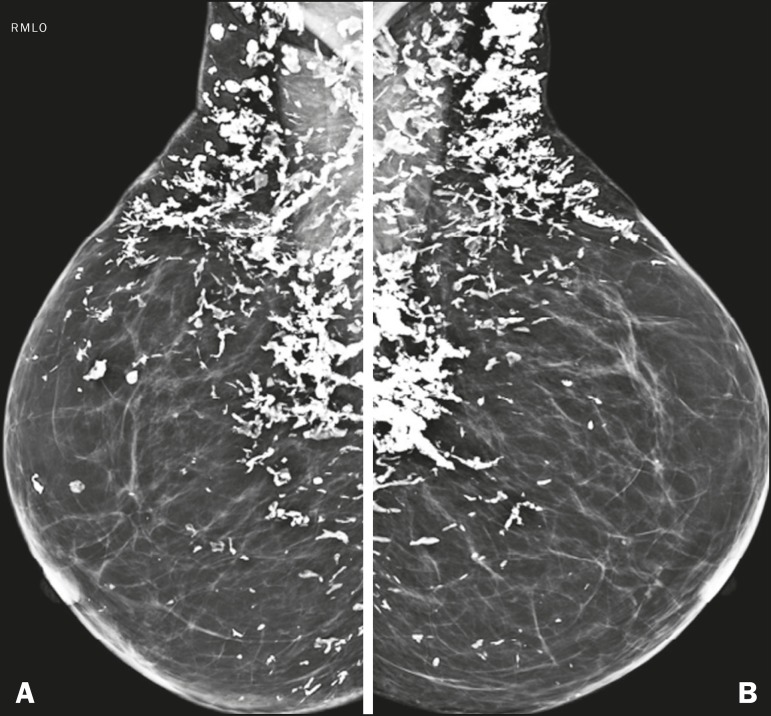
Mammogram, in mediolateral oblique views, of a patient clinically diagnosed with scleroderma, showing several dystrophic calcifications, predominantly in the upper quadrants of the breasts.

**Figure 11:**
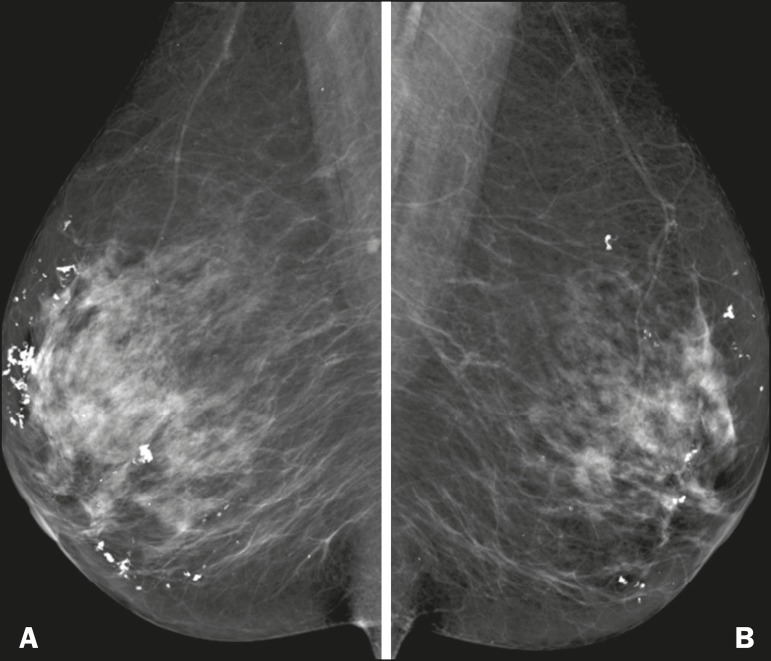
Mammogram of 69-year-old female patient, clinically diagnosed with dermatomyositis, showing bilateral dystrophic calcifications.

Connective tissue diseases are a heterogeneous group of diseases characterized by inflammatory processes in the connective tissues. They include systemic lupus erythematosus, scleroderma, dermatopolymyositis, and mixed connective tissue disease. The most common findings are bilateral axillary lymph node enlargement, skin thickening, and calcifications. In systemic lupus erythematosus, it is common to find skin thickening with multiple subcutaneous nodules, incipient linear calcifications that later become more numerous and coarse, representing areas of fat necrosis^(6,8)^, as can be seen in Figure 9. Scleroderma manifests as thickening of the skin, trabecular thickening of the fibroglandular tissue, and coarse superficial calcifications (Figure 10). Dermatopolymyositis typically presents as cutaneous and dystrophic calcifications (Figure 11).

## LYMPHOMA/LEUKEMIA

**Figure 12:**
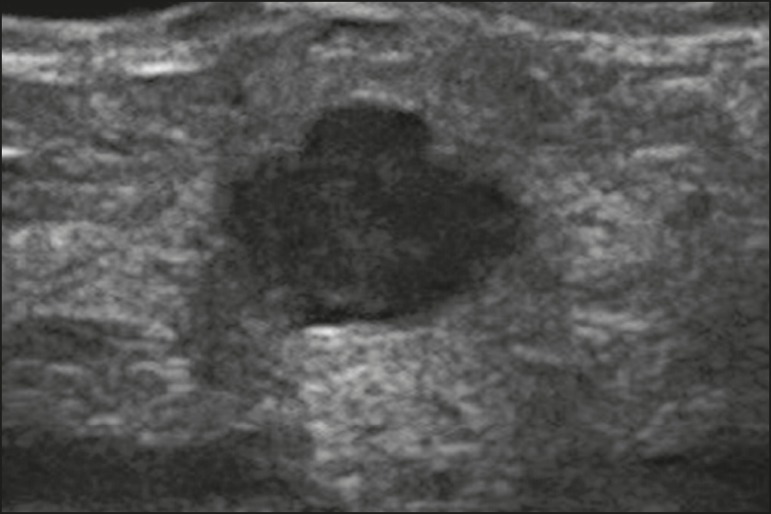
Ultrasound showing a solid, oval, circumscribed, hypoechoic mass with posterior acoustic shadowing, located in the lower inner quadrant of the left breast. Biopsy of the mass led to a diagnosis of B-cell lymphoma.

Secondary involvement of the breasts by lymphoma is uncommon, mainly due to the rarity of lymphoid tissue. Secondary lymphomas are associated with prior or concomitant systemic lymphoma and are more common than primary lymphomas. The most common subtype is diffuse large B-cell non-Hodgkin lymphoma. Secondary lymphomas manifest as masses, as well as focal or global asymmetry. The masses are oval or round, with circumscribed or microlobulated margins (Figure 12), mimicking benign lesions^(7)^.

Leukemic infiltration of the breasts is extremely rare, being most common after bone marrow transplantation. Clinically, there are palpable masses; on mammography, the masses are rounded, microlobulated, and hyperdense, whereas they are hypoechoic or solid-cystic (complex) on ultrasound^(9)^.

## METASTASES

**Figure 13:**
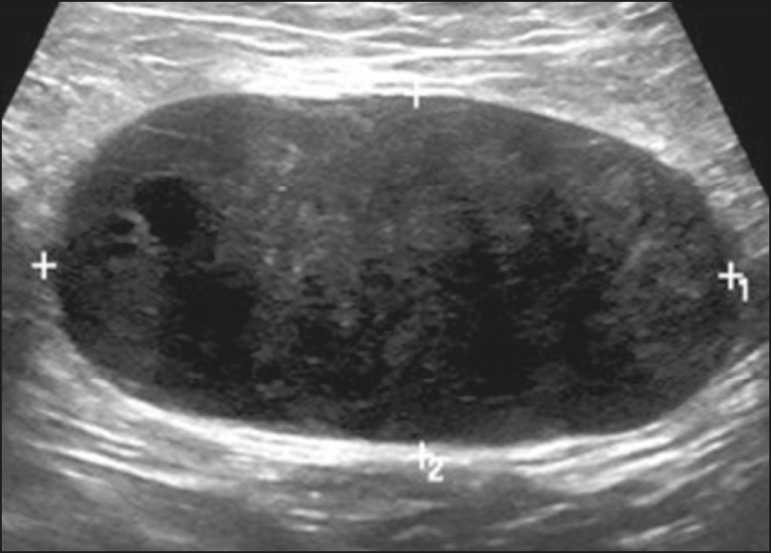
Ultrasound showing an oval, hypoechoic mass with circumscribed margins, its longest axis being parallel to the skin, located in the left axillary tail of the breast. The patient had a history of malignant melanoma. Analysis of a percutaneous biopsy of the mass confirmed the secondary involvement of the breast by melanoma.

**Figure 14:**
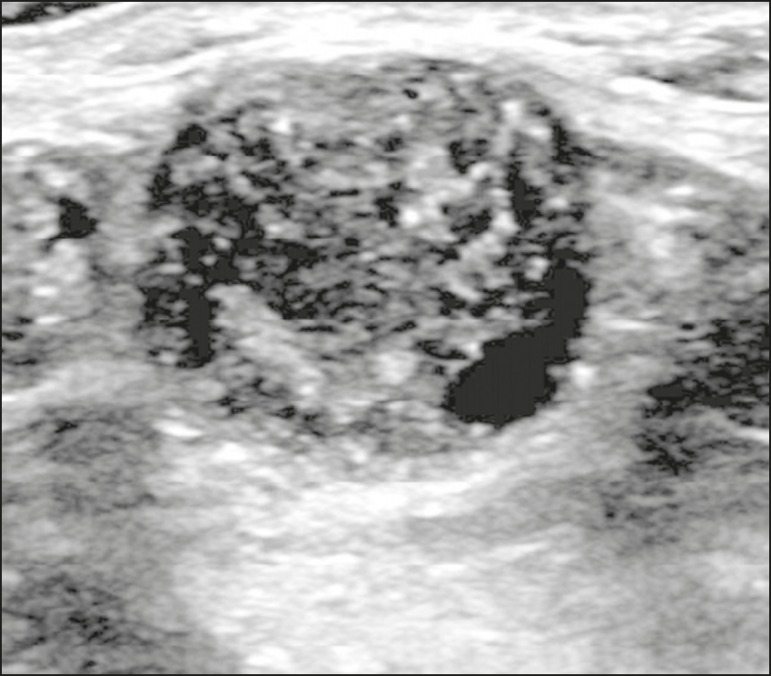
Ultrasound showing a rounded nodule with circumscribed, hypoechoic margins, containing some echogenic foci (calcifications), and posterior acoustic shadowing in the lower inner quadrant of the left breast. Analysis of a percutaneous biopsy of the mass revealed that it was secondary to an ovarian carcinoma.

Secondary lesions in the breast are uncommon, due to the limited arterial supply. The main types of primary cancer are melanoma, thyroid cancer, and ovarian cancer. Mammography shows masses with benign characteristics-oval, circumscribed, and not calcified-as depicted in Figure 13. Ultrasound shows masses that are oval or round, hypoechoic with posterior acoustic shadowing, due to the high cellularity, and presenting as calcifications in ovarian cancer (Figure 14) or thyroid cancer. The nodules are usually located in the superficial planes and are often palpable^(10)^.

## CONCLUSION

Although the breast is not a common site of lesions caused by systemic diseases, its involvement can occur after benign or malignant changes. Knowledge of the main changes found on breast imaging can increase the range of differential diagnoses of an imaging change and occasionally avoid an unnecessary invasive procedure.
